# 

**Published:** 1916-08-26

**Authors:** 


### GIFTS TO MEDICAL MISSIONS.

Miss Theodora A. Roberts, of Florence, Italy, who died on March 19, left £100 to the Medical Missionary Association, Ivory Park, and £3,200 stock in trust for the Florence Medical Mission, and on the death of the survivor of her brother and herself £500 each to the Children's Medical Mission and the Mission to Lepers. Mr. J. W. Lamb, of Nottingham, who died on May 13, left £2,000 to the Baptist Medical Missionary Society.

### Addenbrooke's Hospital, Cambridge.

The accounts for the last quarter, presented at the general quarterly Court of Governors held at Addenbrooke's Hospital on August 14, 1916, showed that there was an increase of about £750 in the expenditure, to a very large extent accounted for by the continual advances in the price of all commodities. The hospital has recently received five legacies, aggregating £900. Furthermore, application was made on behalf of the hospital about two years ago for a share of the Geldart Estate. This estate was left by the late Mrs. Geldart, of Huntingdon, for the benefit of such charitable institutions as the trustees might select. Recently an intimation has been received to the effect that the Public Trustee has been authorised as administrator of the estate to pay to the treasurer of Addenbrooke's Hospital the sum of £1,500 for general purposes, and to pay to the Official Trustees of Charitable Funds the sum of £7,500, to be held as part of the endowment of the hospital, with liberty to apply in building; the income of the investment thereof will be paid to the treasurer for general purposes until the fund is laid out in building. Four-fifths of the £1,500 has already been received, *i.e.* £1,200, and four-fifths of the £7,500 will be handed over by the Public Trustee to the Charity Commissioners in due course.

The committee were glad to be able again to draw attention to the contributions received from the various Friendly Societies' Hospital Sunday Parades. In many cases last year these collections exceeded in amount those of previous years. This year, in several instances (at Littleport, at St. Ives, at Soham, at Little Downham, at Haddenham, at Fordham, at Burwell, at Welney, and at Swaffham Bulbeck), the amount handed over to the hospital is still greater. This is most encouraging to the committee at this time of anxiety as to the financial future of the hospital, and both they and the governors expressed their gratitude to all those who have so successfully organised the collections and rendered the hospital such valuable assistance. They venture to hope that these examples may be an incentive to the friendly societies and committees in other towns and villages to do likewise.

### PASSING EVENTS AND TOPICS.

### Manchester Schools as Hospitals.

Twelve Manchester schools are now under the control of the military authorities as temporary hospitals.

### Nottingham General Hospital's Anniversary Sermon.

The Bishop of Manchester (Dr. Knox) is to preach the anniversary sermon of the Nottingham General Hospital on October 31.

### Australians to Equip Hospital for Russia.

As a tribute from the Commonwealth the people of Australia are raising £60,000 to equip a military hospital for presentation to Russia.

### Infant Welfare Appointment in Leeds.

The Leeds Sanitary Committee has decided to appoint a female medical assistant for infant welfare work in the city, at a salary of £350 per annum.

### Welsh Castle as Red Cross Hospital.

Lord Dunraven has handed over his magnificent Welsh seat, Dunraven Castle, for use as a Red Cross hospital. Between eighty and one hundred beds will be provided.

### Extensions at Stepping Hill Hospital.

A new pavilion, to accommodate 100 beds for wounded soldiers, has been added to the Stepping Hill Hospital, Stockport. Military patients now occupy 439 beds, and a further extension of the hospital is to take place.

### Southport Hospital's Loss.

By the death of Mr. C. W. Bayley, Southport hospitals have lost a valuable friend. He was chairman of the finance committee of the Southport Infirmary and honorary treasurer of the Meols Hall Convalescent Hospital.

### An Example to be Followed.

A Caerphilly lady, Miss Catherine Anthony, has presented the King Edward VII. Hospital, Cardiff, with the unexpired lease of fifty-one years of a fine residence in Newport Road, Cardiff, to be devoted to such purposes as the board of management may determine, in memory of her great-nephew, Second-Lieut. Henry Birrell Anthony, who was killed at Ypres.

### Health Committee and the Rise in Prices.

A request from the Merthyr Health Committee for an extra 5s. per week for each tuberculosis patient at the Mardy Isolation Hospital, on the ground of the increased price of food and commodities, has been refused by the Welsh National Memorial Association.

### University College Hospital Medical School.

Since the commencement of the war past and present students of University College Hospital and Medical School numbering 615 have joined the Navy and Army. Of these eleven have died in the service of the country. The following honours have been gained :—K.C.M.G., 1; C.M.G., 7; C.B., 5; V.C. (Clasp), 1; D.S.O., 6; Legion of Honour (Croix de Commandeur), 1; mentioned in dispatches, 53 (7 twice).

### A Review of Women V.A.D.s.

Five Hundred members of the Leicestershire Women's V.A.D. were reviewed on the football ground at Leicester recently by Colonel Astley Clarke, A.D.M.S. They looked very smart on parade and gave a demonstration afterwards which showed them to be thoroughly efficient in their work. They came in for some highly complimentary remarks from the Duke of Rutland, who commented upon the extraordinary development of the V.A.D. movement.

### A War Memorial.

Speaking at the Prize Day of the Wolverhampton Royal Orphanage, whose year finished with a deficiency of nearly £700, the Earl of Dartmouth pointed out one way in which the problem of keeping such institutions adequately supplied with funds during war-time could be solved. Mr. and Mrs. George W. Hayes, of Worplesdon Hill, near Woking, had forwarded 700 guineas to the institution for the foundation of a bed, for the reception of a child from the Chester district, in memory of their son, a subaltern of the Black Watch, who was killed in action in France. A provision of this kind is a nobly conceived tribute to a gallant son.

### THE WELLCOME HISTORICAL MEDICAL MUSEUM.

The Wellcome Historical Medical Museum will be closed for cleaning from September 1 to 30 inclusive. It will reopen in October with an interesting loan exhibition illustrating the Folk-Lore of London, including medical charms, amulets, and other objects used in connection with the cure and prevention of disease.

### GALLANT R.A.M.C. OFFICERS.

A further list of awards for gallantry, issued by the War Office on August 19, contains the names of a number of officers of the Royal Army Medical Corps and the Canadian Army Medical Corps whose gallantry and devotion to duty have received recognition. Temporary Captain E. C. Gimson and Temporary Captain P. L. Watkin-Williams each receive the D.S.O. for conspicuous gallantry when attending the wounded. The Military Cross has been awarded to each of the following: Temporary Lieutenant G. P. Armstrong, M.D.; Temporary Lieutenant F. R. Hassard, M.B.; Temporary Captain H. A. Pallant; Temporary Lieutenant V. F. Stock, M.B.; and Temporary Lieutenant G. B. Wiswell, M.D. All the above gallant officers were members of the Royal Army Medical Corps. The Military Medal has also been awarded to each of the following officers of the Canadian Army Medical Service: Captain C. W. Johnston, Captain W. J. McAlister, Captain J. B. McGregor, Captain A. Ross, Captain F. J. Tees, and Captain H. W. Wadge.

**Rates of Subscription:** Twelve months, 6/6; Six months, 4/-; Three months, 2/-, post free to any address in the United Kingdom. Abroad, Twelve months, 8/8; Six months, 5/-; Three months, 2/6, post free. The Hospital "Index" to Volume LIX., October 1915 to March 1916, is now ready, price 1½d. per copy, post free. Address The Manager, "The Hospital," The Hospital Building, 28/29 Southampton Street, Strand, London, W.C.

